# ERK5 signalling pathway is a novel target of sorafenib: Implication in EGF biology

**DOI:** 10.1111/jcmm.16990

**Published:** 2021-10-16

**Authors:** Marta Ortega‐Muelas, Olga Roche, Diego M. Fernández‐Aroca, José A. Encinar, David Albandea‐Rodríguez, Elena Arconada‐Luque, Raquel Pascual‐Serra, Ismael Muñoz, Isabel Sánchez‐Pérez, Borja Belandia, María J. Ruiz‐Hidalgo, Ricardo Sánchez‐Prieto

**Affiliations:** ^1^ Laboratorio de Oncología Molecular Unidad de Medicina Molecular Centro Regional de Investigaciones Biomédicas Universidad de Castilla‐La Mancha Unidad Asociada de Biomedicina UCLM Unidad asociada al CSIC Albacete Spain; ^2^ Departamento de Ciencias Médicas Facultad de Medicina Universidad de Castilla‐La Mancha Albacete Spain; ^3^ Instituto de Investigación Desarrollo e Innovación en Biotecnología de Elche (IDiBE) e Instituto de Biología Molecular y Celular (IBMC) Universidad Miguel Hernández (UMH) Elche Spain; ^4^ Departamento de Biología del Cáncer Instituto de Investigaciones Biomédicas ‘Alberto Sols’ (CSIC‐UAM) Unidad asociada de Biomedicina UCLM Unidad asociada al CSIC Madrid Spain; ^5^ Departamento de Bioquímica Facultad de Medicina Instituto de Investigaciones Biomédicas ‘Alberto Sols’ (CSIC‐UAM) Unidad asociada de Biomedicina UCLM Unidad asociada al CSIC Madrid Spain; ^6^ Área de Bioquímica y Biología Molecular. Facultad de Medicina Universidad de Castilla‐La Mancha Albacete Spain; ^7^ Instituto de Investigaciones Biomédicas ‘Alberto Sols’ Consejo Superior de Investigaciones Científicas (IIBM‐CSIC)–Universidad de Castilla‐La Mancha (UCLM) Albacete Spain

**Keywords:** EGF, ERK5, MEK5, Sorafenib

## Abstract

Sorafenib is a multikinase inhibitor widely used in cancer therapy with an antitumour effect related to biological processes as proliferation, migration or invasion, among others. Initially designed as a Raf inhibitor, Sorafenib was later shown to also block key molecules in tumour progression such as VEGFR and PDGFR. In addition, sorafenib has been connected with key signalling pathways in cancer such as EGFR/EGF. However, no definitive clue about the molecular mechanism linking sorafenib and EGF signalling pathway has been established so far. Our data in HeLa, U2OS, A549 and HEK293T cells, based on in silico, chemical and genetic approaches demonstrate that the MEK5/ERK5 signalling pathway is a novel target of sorafenib. In addition, our data show how sorafenib is able to block MEK5‐dependent phosphorylation of ERK5 in the Ser218/Tyr220, affecting the transcriptional activation associated with ERK5. Moreover, we demonstrate that some of the effects of this kinase inhibitor onto EGF biological responses, such as progression through cell cycle or migration, are mediated through the effect exerted onto ERK5 signalling pathway. Therefore, our observations describe a novel target of sorafenib, the ERK5 signalling pathway, and establish new mechanistic insights for the antitumour effect of this multikinase inhibitor.

## 1 INTRODUCTION

Sorafenib has been shown to be a potent multikinase inhibitor. Initially proposed as an inhibitor of the MAP3K for ERK1/2, Raf, latter on it was shown to block key tyrosine kinase receptors in tumour progression such as platelet‐derived growth factor receptor (PDGFR) and vascular endothelial growth factor receptor (VEGFR).^1^ Indeed, the number of pathologies where sorafenib has become a key component of the therapeutic armamentarium has increased widely, and includes renal carcinoma, hepatocellular carcinoma or differentiated thyroid cancer, among others (for a review^2^). In this sense, EGF signalling pathway, a key pathway in cancer, has been connected with sorafenib in terms of resistance.^3^, ^4^, ^5^ In fact, new compounds derived from sorafenib act through EGFR.^6^ Furthermore, treatment combining EGFR inhibitors and sorafenib has been considered, showing promising results in lung cancer.^7^, ^8^ In addition, it is also noteworthy that sorafenib is known to affect key biological processes in cancer progression as, for example, cell viability, motility or invasion,^9^, ^10^ which are also triggered by EGF signalling (for a review see Ref. [11, 12]). Interestingly, both EGFR activation and sorafenib seem to affect in opposite ways the MAPKs‐mediated signalling. The MAPKs, a family of serine/threonine kinases, is one of the best‐characterized signalling pathways in cancer with important therapeutic implications (for a review see Ref. [13]). In fact, it is very well established that Raf, the original target of sorafenib,^14^ is a critical mediator in the biological properties associated with the activation of EGFR^15^ as well as in its oncogenic properties.^16^ In addition, EGF is a classical stimulus to activate several MAPKs such as ERK1/2 and ERK5.^17^, ^18^ Interestingly, it has been shown that sorafenib is able to block ERK1/2 in different experimental models.^19^, ^20^ Although no direct relationship between sorafenib and ERK5 has been demonstrated, it has been recently shown how oncogenic forms of B‐Raf regulate ERK5 activity.^21^ Moreover, it is known that ERK5 is implicated in the therapeutic combination of Raf and MEK inhibitors.^22^

Considering all the previous, we decided to study the role of the MAPKs ERK1/2 and 5 in the putative effects of sorafenib onto EGF signalling. Our data demonstrate that the ERK5 signalling pathway is a novel target of sorafenib in response to EGF. This effect is exerted, at least, onto the MAP2K MEK5, which in turn, blocks the activation of ERK5. This inhibitory effect is critical to explain the implication of sorafenib in different processes such as progression through cell cycle or migration. Our observations could be a novel explanation for some of the therapeutic benefits associated with sorafenib in cancer therapy and open the possibility to include new pathologies with an aberrant ERK5 signalling pathway in sorafenib‐based therapy.

## 2 MATERIALS AND METHODS

### 2.1 Cell lines and plasmids

HeLa, U2OS, A549 and HEK293T cell lines were purchased from ATCC and maintained in 5% CO_2_ and 37°C. Cells were grown in Dulbecco's modified Eagle's medium supplemented with 10% foetal bovine serum (FBS), 1% glutamine plus antibiotics. All cell culture reagents were provided by Lonza.

Plasmids pCEFL HA‐ERK5KD, pCEFL HA‐ERK5WT and pCEFL MEK5DD have been previously described.^23^ pLKO.1‐shRNA ERK5 (TRCN0000010275) and pLKO.1‐empty vector were obtained from Sigma‐Aldrich.

Plasmid pSLIK MEK5DD‐mRFP1 was a gift from Dr. Kevin Janes (Addgene plasmid #47548; http://n2t.net/addgene:47548 RRID:Addgene_47548.^24^; Plasmid pRL Renilla Luciferase Control Reporter Vector was obtained from Promega (E2231), and plasmid pGL3‐3XMEF2‐luc was a gift from Dr. Ron Prywes (Addgene plasmid #32967; http://n2t.net/addgene:32967; RRID: Addgene_32967).

### 2.2 Chemicals and antibodies

ERK5, phospho‐ERK5 (Ser218/Tyr220), phospho‐ERK1/2 (Thr202/Tyr204) and phospho‐SGK1 (Ser78) antibodies were purchased from Cell Signaling Technology. Vinculin and ERK2 antibodies were purchased from Sigma‐Aldrich and Santa Cruz Biotechnology, respectively. HA antibody was obtained from BioLegend.

ERK5 inhibitor XMD8‐92, sorafenib and MEK1/2 inhibitor U0126 (Selleckchem) were dissolved in DMSO and stored at −20°C until used. EGF (Sigma‐Aldrich) and Doxycycline (Merck) were dissolved in double‐distilled water, aliquoted and stored at −20°C until used.

### 2.3 Molecular docking simulations

The crystallographic structure of the catalytic domain of the human proteins B‐Raf (UniProt code: P15056, BRAF_HUMAN, PDB code: 6U2G), MEK1 (UniProt code: Q02750, MP2K1_HUMAN, PDB code: 5HZE), MEK2 (UniProt code: P36507, MP2K2_HUMAN, PDB code: 4H3Q), ERK2 (UniProt code: P28482, MK01_HUMAN, PDB code: 6G97) and ERK5 (UniProt code: Q13164, MK07_HUMAN, PDB code: 4IC7) have been obtained from the Research Collaboratory for Structural Bioinformatics (RCSB) Data Bank (PDB). However, these proteins have amino acid regions that are not resolved from the crystallographic data and even side chains of certain amino acids are missing. Therefore, all of them have been modelled by homology using as a template the structure chosen in each case for each protein. This modelling has been done through the Swiss‐Model web application,^25^ in automatic mode. Thus, both docking and molecular dynamics simulations are performed on structures that do not present ‘unresolved gaps’, which would otherwise alter the interpretation of results. For the MEK5 protein (UniProt Code: Q13163, MP2K5_HUMAN), the structure of its catalytic domain has not yet been solved, so we have carried out homology modelling in the Swiss‐Model web application,^25^ using as template the structure 3ZLS, resolved for MEK1, with which it shares a sequence identity of 47.65%. Specific editing of these protein structures has been carried out using PyMol software (PyMOL Molecular Graphics System, v2.3.3 Schrödinger, LLC, at http://www.pymol.org/), without further optimization.

Molecular docking simulations of sorafenib on the structures of the aforementioned protein kinases have focused on its ATP‐binding site in the catalytic region. They have been carried out using the YASARA Structure v19.12.14 software, as an interface that executes AutoDocK 4 as docking software and the AMBER99 force field^26^ has been used. A total of 999 sorafenib dockings have been launched for calculation, considering their possible conformers by rotations between atoms and the variation of Gibbs free energy (ΔG, kcal/mol) has been calculated for each result. The results have been clustered when the distance between different conformers of the docked ligand has been less than 7 Å. The YASARA software allows a control of the pH, which has been established at 7.4 in all simulations. Autodock uses a force field function to prioritize each conformer that considers the strength of electrostatic interactions, hydrogen bridging, van der Waals interactions, and also contributions from solvation and entropy.^27^

### 2.4 Molecular dynamics simulations

All molecular dynamics simulations were executed with the YASARA Structure v19.12.14 software, and the AMBER14 force field was used. During the simulation, the cuboid cell was allowed to include 20 Å around the protein and was filled with water at a density of 0.997 g/ml. The initial energy minimization has been performed under relaxed constraints, using the steepest descent minimization. The simulations were carried out under constant pressure and temperature conditions (1 atm and 25°C). To mimic physiological conditions 0.9% NaCl has been added. The pH has been maintained at 7.4, and hydrogen atoms were added to the protein structure in the appropriate ionizable groups according to the calculated pKa relative to the simulation pH (ie one hydrogen atom is added if the calculated pKa is greater than the pH). The pKa has been calculated for each residue according to the Ewald method.^28^ All the steps of each simulation have been executed by a pre‐installed macro (md_run.mcr) within the YASARA suite. Data have been recorded every 100 ps. The values of solvation binding energy or Molecular Mechanics/Poisson‐Boltzmann surface area (MM/PBSA) have been calculated using the YASARA macro md_analyzebindenergy.mcr.^29^

### 2.5 Transfections and infections

Sub‐confluent cultures of HEK293T cells, in 10 cm plates, were transfected using the calcium phosphate technique following standard procedures with 2 µg of pCEFL HA‐ERK5WT or ERK5KD in the presence or absence of 10 µg of pCEFL MEK5DD. Total amount of DNA was normalized using pCEFL empty vector. Eight hours later, transfection media were replaced with cell culture media until samples were treated and collected.

Lentiviral production and cell infection were performed as previously described.^20^, ^30^ Briefly, HEK293T cells were transfected overnight by using calcium phosphate with 9 µg of PLKO.1‐shRNA ERK5, PLKO.1‐empty vector or pSLIK MEK5DD‐mRFP1, plus 6 µg of PSPAX2, and 3 µg of the viral envelope protein, VSVG. Supernatants were collected 48 hours after transfection and added to the cells for 16 hours in the presence of 8 µg/ml polybrene. Forty‐eight hours post‐infection, cells expressing the shRNAs were selected with puromycin (Sigma‐Aldrich) for 72 hours. Each experiment was performed with at least two different pools of infection.

### 2.6 Western blotting

Cells were collected in lysis buffer (HEPES 100 mM, Triton‐X100 0.8%, NaCl 5 mM, EDTA 5 mM and EGTA 5 mM). Protease and phosphatase inhibitors (Sigma‐Aldrich) were added prior to lysis. Protein quantification was performed by using the BCA Protein Assay Kit (Thermo Fisher) following the manufacturer's instructions. Indicated amounts of protein were loaded onto appropriate percentage SDS‐PAGE, transferred to PVDF membranes with the semi‐dry Pierce Power Blot (Thermo Fisher) and blotted against different proteins using specific antibodies.

Antibody detection was achieved by enhanced chemiluminescence (Amersham) in a LAS‐3000 system (FujiFilm). Results show a representative blot out of three with nearly identical results. Vinculin was used as a loading control. Band quantification was performed by using UN‐SCAN‐IT Graph Digitizer software (Silk Scientific). Images show a representative experiment out of three with nearly identical results.

### 2.7 RNA isolation, reverse transcription and real‐time quantitative PCR

Total RNA was obtained, and reverse transcription was performed as previously described.^30^ cDNA synthesis was performed with RevertAid First Strand cDNA synthesis Kit (Thermo Scientific) following manufacturer's protocol in an iCycler thermal cycler (Biorad). Real‐time PCR was performed with Fast SYBR Green Master kit (Thermo Scientific) in a 7500 Fast Real‐Time PCR instrument (Applied Biosystems). The PCR conditions were performed as previously described.^30^ Primers were designed by using the NCBI BLAST software and purchased from Sigma‐Aldrich. Primers sequences used are as follows: ERK5 forward 5′‐AGCACTTTAAACACGACAAC‐3′; ERK5 reverse 5′‐ TAGACAGATTTGAATTCGCC‐3; GAPDH forward 5′‐TCGTGGAAGGACTCATGACCA‐3′; GAPDH reverse 5′‐CAGTCTTCTGGGTGGCAGTGA‐3′.

### 2.8 Thymidine block and flow cytometry procedure

Thymidine block was performed as previously described.^31^ Briefly, cells were seeded in 10 cm plates at 20%–30% confluence. Thymidine (Sigma‐Aldrich) was added to a final concentration of 2 mM 16 h after plating and incubated for 18 h. Next, thymidine was removed by washing with sterile PBS 1X. Then, cells were re‐incubated with fresh medium for 9 h. Finally, a second round of thymidine was added at a 2 mM concentration for another 18 h. Finally, cells were trypsinized and collected for cell cycle analysis as previously described.^30^ Briefly, cells were fixed with cold 70% ethanol in PBS at 4°C and extensively washed with cold PBS. The cells were then incubated with 10 µg/ml propidium iodide (PI) and 20 µg/ml RNase for 20 min in darkness. Samples were analysed in a MACSQuant Analyzer 10 (Miltenyi Biotec). Data were analysed by using Flowing Software (University of Turku).

### 2.9 Reporters assays

HeLa cells were transfected with Lipofectamine LTX (Invitrogen) according to the manufacturer's instructions. For the transient transcriptional assays, 4x10^4^ cells per well were seeded in 24‐well plates, 24 h prior to transfection. Fifty ng of the reporter gene luciferase (3XMEF2‐LUC), 100 ng of pCEFL MEK5DD and 100 ng of pCEFL HA‐ERK5WT were transfected. As an internal control of transfection efficiency, 5 ng of a vector containing the reporter gene renilla‐luciferase was used. The amount of total DNA transfected at all points in an experiment was matched with pCEFL empty plasmid. After 6 h, the DNA‐Lipofectamine LTX complexes were removed, and fresh medium with the corresponding treatments was added for 16 h before collecting the cells for further processing. The luciferase and renilla activities were determined using the Dual‐Luciferase® Reporter Assay System kit (Promega) in a GLoMAX^®^ luminometer (Promega) according to the manufacturer's instructions. The luciferase activity was normalized by the renilla activity to correct the transfection efficiency between samples. The results represented for each experimental point refer to the luciferase/renilla ratio relative to the activity obtained in the transfected cells with pCEFL empty vector.

### 2.10 Migration assays

For migration assays, 2 × 10^4^ cells were seeded on the upper compartment of 8 µm‐pore transwells (Corning Incorporated) in FBS‐free culture medium. FBS‐containing culture medium was added to the lower compartment. After 24 h, cells on the upper surface of the transwell were removed using a cotton swab and cells attached to the lower surface were stained with Diff‐Quik reagent (Dade Behring). Images of stained cells (7 fields/transwell) were captured with a Zeiss LSM800 confocal laser microscope and migrating cells were counted. Images were processed using ImageJ plugin ‘Cell counter’.

### 2.11 Statistical analysis

Data are presented as mean ±standard deviation (SD) of at least 3 independent experiments. Statistical significance was evaluated by Student's t test in GraphPad Prism v7.0 software. Also, for molecular dynamics results, a two‐way ANOVA statistical analysis has been performed and group pairwise differences have been detected using Dunnet's test. The statistical significance of differences is indicated in figures by asterisks as follows: *p < 0.05, **p < 0.01 and ***p < 0.001.

## 3 RESULTS

### 3.1 Sorafenib blocks ERK5 activation mediated by EGF through MEK5 inhibition

**FIGURE 1:**
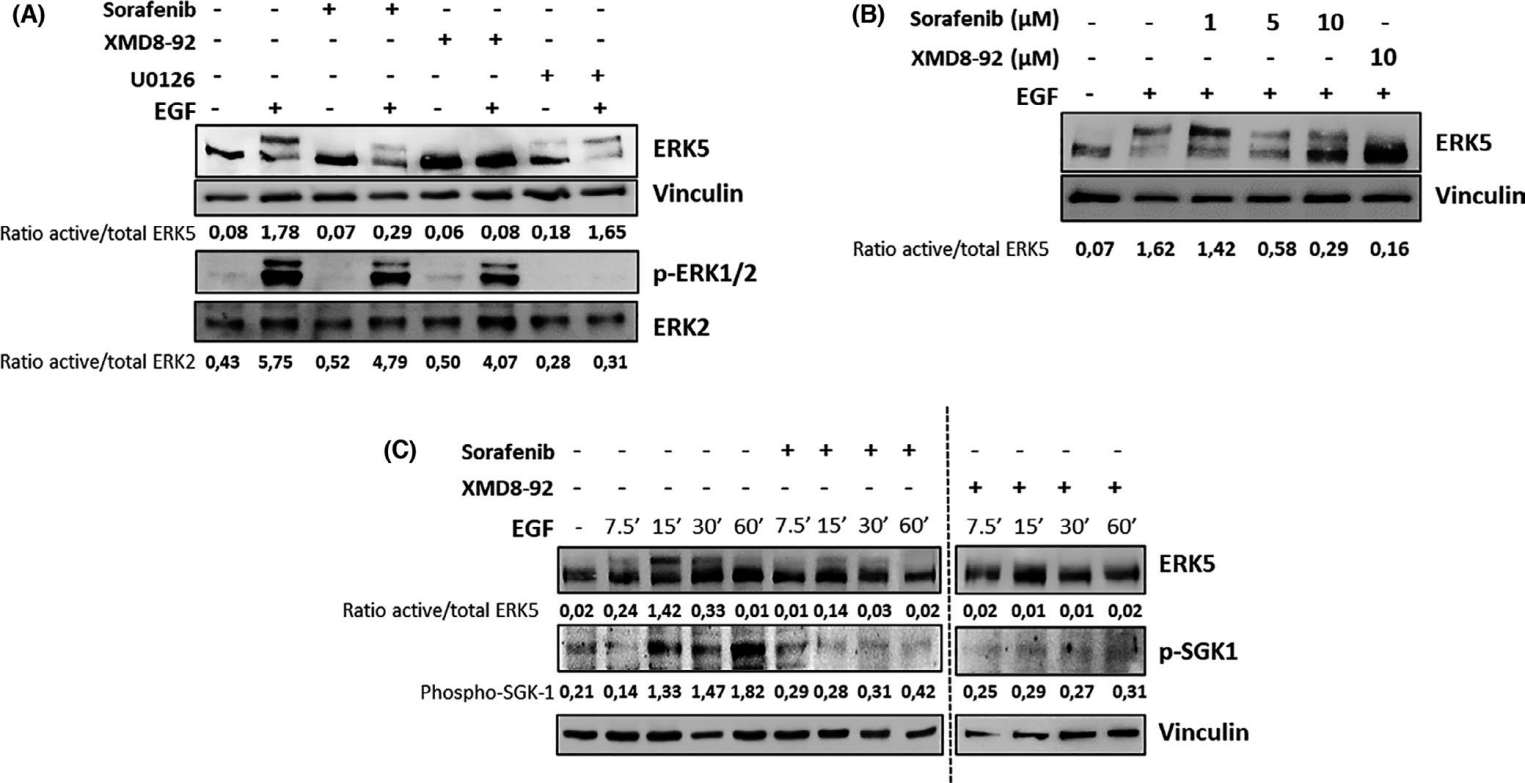
Sorafenib blocks ERK5 activation mediated by EGF in HeLa cells. (A) Sub‐confluent cultures of HeLa cells were exposed to sorafenib (10 µM), XMD8‐92 (10 µM) or U0126 (10 µM) for 30 minutes and then exposed for 15 minutes to 2 ng/ml EGF. Then, total cell lysates were collected and protein extracts (60 µg for ERK1/2 or 120 µg for ERK5) were blotted against the indicated antibodies. Vinculin was used as a loading control. (B) Sub‐confluent cultures of HeLa cells were exposed to sorafenib at the indicated concentrations for 30 minutes and then exposed for 15 minutes to 2 ng/ml EGF. Then, total cell lysates were collected, and protein extracts (120 µg) were blotted against the indicated antibodies. As a control, cells were exposed to 10 µM XMD8‐92 in the same conditions. Vinculin was used as a loading control. (C) Sub‐confluent cultures of HeLa cells were exposed to sorafenib (10 µM) or XMD8‐92 (10 µM) for 30 minutes and then exposed to 2 ng/ml EGF for the indicated times. Then, total cell lysates were collected, and protein extracts (120 µg) were blotted against the indicated antibodies. Vinculin was used as a loading control. Numbers below blots indicate the ratio between active and total protein, except for the case of p‐SGK1 that shows intensity of the bands normalized by the loading control. Images show a representative blot out of 3 with nearly identical results

It is known that EGF promotes a marked activation of different members of the MAPK family as ERK1/2 and ERK5. Therefore, we challenged the effect of sorafenib onto the activation of these two MAPKs mediated by EGF in our model of HeLa cells. Treatment with EGF promotes a marked activation of both ERK1/2 and ERK5, which was blocked by specifics inhibitors of these MAPKs signalling pathways as U0126 or XMD8‐92 (Figure 1A). Interestingly, pre‐treatment of HeLa cells with sorafenib for 30 minutes only affects ERK5 activation, as judged by the motility shift of the protein as well as the bands quantification, with a minimum effect onto ERK1/2 (Figure 1A). Furthermore, our observation was challenged in other experimental models, such as U2OS and A549 cell lines (Figure S1), showing similar results than in HeLa cells in terms of ERK5 inhibition. Next, we challenged the effect of sorafenib in a dose‐response assay. As it is shown, Figure 1B, sorafenib promotes a marked reduction of ERK5 activation after EGF exposure in a dose‐dependent fashion, with a reduction above 80% at 10 μM of EGF. As a control, incubation with XMD8‐92 at 10 μM was used, showing almost a complete blockage of ERK5 activation by EGF. In addition, a time‐course experiment was performed showing a marked inhibitory effect of sorafenib in ERK5 activation after EGF exposure in all time points selected (Figure 1C). Finally, we decided to prove our observation about the inhibitory effect of sorafenib onto the ERK5 signalling pathway not only by analysing the motility shift. Therefore, we took advantage of the availability of antibodies against a well‐established substrate of ERK5, such as the Ser78 of SGK1.^32^ As it is shown in Figure 1C, sorafenib almost abolished the phosphorylation of SGK1 at Ser78 in response to EGF in a similar fashion to XMD8‐92. In sum, all these evidences indicate that the ERK5 signalling pathway is a novel target of the multikinase inhibitor sorafenib.

**FIGURE 2:**
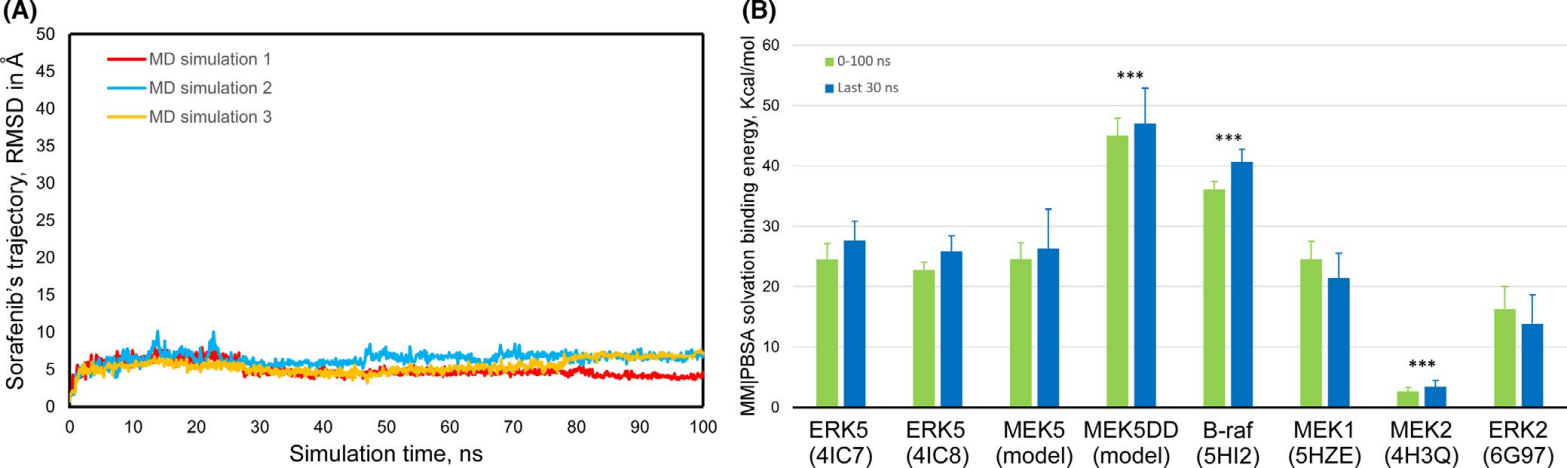
Analysis of molecular dynamics simulations for catalytic domain structures of different kinases with sorafenib docked to its ATP‐binding site. Panel A shows an example of trajectory analysis of sorafenib bound to the ATP‐binding site in MKK5 kinase during a 100 ns molecular dynamics simulation. Panel B shows measurements of solvation binding energy of sorafenib bound to the ATP‐binding site of different kinases (the structure used for each protein kinase is indicated). The more positive values indicate greater affinity of the compound for each kinase. Average values ±standard deviation (n = 3) are shown

To fully address this interesting observation, we decided to study which could be the potential mechanism. Therefore, to support the role of sorafenib as a putative inhibitor of the MEK5/ERK5 signalling pathway, in silico molecular docking and dynamics simulations studies were performed. To this end, we used ERK5, MEK5 and a constitutive active form of MEK5 (MEK5DD) that mimic the phosphorylated protein under physiological conditions by changing residues 311 and 315 to Aspartic acid,^33^ as well as other related kinases as MEK1, MEK2 and ERK2. The human genome encodes for 538 protein kinases that transfer the γ‐phosphate group from ATP to Ser/Thr (67%) or Tyr (17%)^34^ and that share the secondary structure of their catalytic domain (Figure S2A and S2C). Figure S2B and S2D show the calculated values of Gibbs free energy variation (ΔG, kcal/mol) and the calculated KD, respectively, for different compounds, described as ERK5 inhibitors,^35^, ^36^, ^37^ and sorafenib. Except for AX‐15836, the remaining compounds present a very similar ΔG value compared to the seven protein kinases analysed (−9 ± 1 kcal/mol). Figure S2D presents the calculated KD values without showing clear differences in the affinity of sorafenib for the different kinases (≈1 µM), on which it could behave as a competitive inhibitor for ATP. While molecular docking^38^ generates a ‘static image’ of the binding site on the protein and the conformation of the bound compound, the molecular dynamics simulations take into account the overall behaviour of the system, including ligand, receptor, molecules of water, salt ions, temperature and pressure^39^ throughout the simulation time. Figure 2 shows the results obtained by molecular dynamics simulations of different protein‐kinase‐sorafenib complexes (docked to the ATP‐binding site) that have elapsed during a period of 100 ns. As an example, Figure 2A presents the trajectory of movement of sorafenib, docked to the ATP‐binding site, in triplicate simulations and where we can see that the inhibitor has always remained bound throughout the simulation time (identical behaviour of each inhibitor tested against the different kinases, data not shown). Figure 2B shows the solvation binding energy values (MM|PBSA)^40^ of sorafenib bound to different kinases and distinguishes whether the measurement has been taken throughout the 100 ns of simulation or in the last 30 ns of the simulation. The statistical analysis carried out indicates that, although there are no statistically significant differences between measuring the parameter during the entire simulation or in the last 30 ns, there are significant differences in the solvation binding energy of sorafenib to different enzymes. The constitutively active ME5K (MEK5DD) presents the highest value (47,023 ± 5,879 kcal/mol), even higher than that of B‐Raf with 40,668 ± 2,083 kcal/mol. On the contrary, it is practically negligible for MEK2 (3,455 ± 1,011). These results support that sorafenib is a blocker of MEK5‐ERK5 signalling pathway, preferably of the MEK5 activated form as the data with MEK5DD suggest.

**FIGURE 3:**
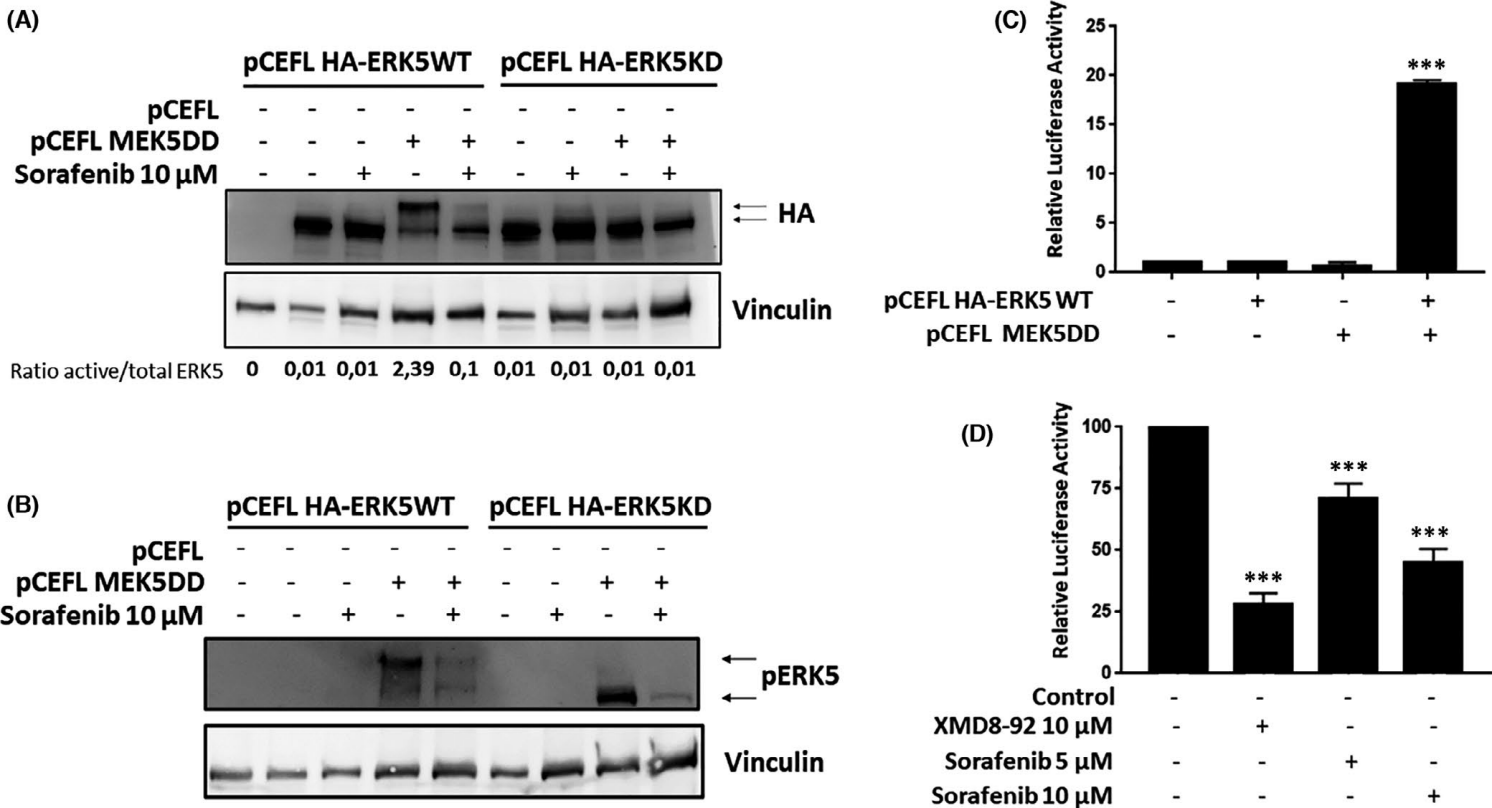
Sorafenib blocks ERK5 activation mediated by constitutive active MEK5. (A) HEK293T cells were transfected by calcium phosphate with 2 µg of PCEFL HA‐ERK5WT or PCEFL HA‐ERK5KD in the presence or absence of 10 µg of pCEFL MEK5DD. Thirty‐six hours later cells were exposed to the indicated dose of sorafenib for 6 h, and samples collected and processed for Western blot and blotted against HA (upper panel). Vinculin (lower panel) was used as loading control for total cell lysates. Images show a representative blot out of 3 with nearly identical results. (B) Same as in A, but using an antibody against active ERK5. Vinculin was used as loading control for total cell lysates. Images show a representative blot out of 3 with nearly identical results. (C) Luciferase assay for HeLa cells transfected with 3XMEF2‐LUC (50 ng) plus renilla (5 ng) in the presence or absence of pCEFL HA‐ERK5WT (100 ng), pCEFL MEK5DD (100 ng) or both. Histogram shows the average of 3 independent experiments evaluating luciferase activity normalized by renilla. Control cells were considered as 1. Bars mean standard deviation. *p < 0.05, **p < 0.01 and ***p < 0.001. (D) Luciferase assay evaluated in HeLa cells co‐transfected with PCEFL HA‐ERK5WT and pCEFL MEK5DD and then incubated in presence/absence of indicated doses of XMD8‐92 or sorafenib for 16 h. Histogram shows the average of 3 independent experiments evaluating luciferase activity normalized by renilla. Transfected but untreated cells were considered as 100. Bars mean standard deviation. *p < 0.05, **p < 0.01 and ***p < 0.001

In light of these in silico evidences and our previous observation showing a lack of motility shift in ERK5, we considered the possibility that sorafenib affects directly the ERK5 signalling pathway at least through inhibition of the MEK5 kinase activity onto ERK5. Therefore, to fully prove this possibility we switched to a transient approach in HEK293T cells using a HA‐tagged ERK5 wild type (HA‐ERK5WT) and a mutant form of HA‐tagged ERK5, unable to render autophosphorylation (HA‐ERK5KD). Then, HEK293T cells were transfected with HA‐ERK5WT or HA‐ERK5KD in the presence/absence of an active MEK5 (MEK5DD). As it is shown (Figure 3A), a marked increase in HA‐ERK5WT activation was observed in the presence of MEK5DD as judged by motility shift in total cell lysates as well as band quantification. In addition, the same cell lysates were exposed to an antibody that recognizes specifically MEK5 phosphorylation onto ERK5 (Thr218/Tyr220), showing a robust signal in the presence of MEK5DD (Figure 3B). As expected, the presence of sorafenib was able to abolish the motility shift as well as the signal of ERK5 phosphorylated by MEK5 (Figure 3A and 3B) for HA‐ERK5WT in the presence of MEK5DD. In the case of an inactive ERK5 (HA‐ERK5KD), no motility shift was detected in total cell lysates compared to ERK5WT and, consequently, no effect of sorafenib was observed (Figure 3A). However, in terms of ERK5 phosphorylation, the presence of sorafenib clearly diminished the intensity of the band corresponding to HA‐ERK5KD phosphorylated by MEK5DD (Figure 3B). Therefore, these data support a direct effect of sorafenib onto ERK5 phosphorylation mediated by MEK5. Next, we challenged the effect of sorafenib on transcriptional reprogramming associated with exclusive ERK5 activation. To this end, HeLa cells were transfected with a plasmid coding a luciferase firefly gene under the control of MEF2 response elements in the presence/absence of HA‐ERK5WT, MEK5DD or both. While transfection of active MEK5 or ERK5 did not promote luciferase activity, the combination of both transgenes showed a prominent increase in luciferase activity, indicating the necessity of the expression of both genes in the following experiments (Figure 3C). Next, we analysed the effect of sorafenib in the luciferase activity in our model of HeLa cells. As it is shown (Figure 3D), constitutive activation of ERK5, promoted a marked increase in luciferase activity that was blocked by the presence of sorafenib in a dose‐dependent fashion.

Therefore, these data support a direct effect of sorafenib in the ERK5 activation mediated by MEK5, indicating that ERK5 signalling pathway is a novel target of sorafenib.

### 3.2 Sorafenib blocks entry in S phase after EGF stimulation or ERK5 activation

**FIGURE 4:**
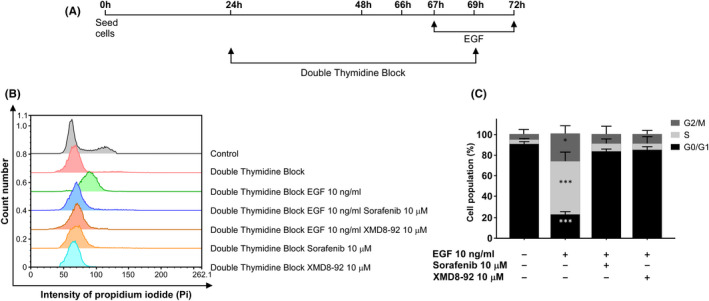
Sorafenib blocks EGF‐mediated S phase induction. (A) Timeline of the experiment. HeLa cells were exposed to double‐thymidine block as indicated in material and methods. Cells were treated with sorafenib or XMD8‐92 at the indicated concentrations during the last 3 h before withdrawal of thymidine block and maintained for 3 h more until the end of the experiment. EGF was added one hour after sorafenib or XMD8‐92 treatment and was also maintained until the end of the experiment. After treatments, cells were collected and evaluated by flow cytometry for cell cycle progression. (B) Graphical representation of cell cycle profile in HeLa cells control and synchronized by using a double‐thymidine block at the different conditions indicated in the graph. (C) Histogram showing the average of three independent experiments representing the percentage of population in the different phases of the cell cycle. Statistics were referred to untreated cells. Bars mean standard deviation. *p < 0.05, **p < 0.01 and ***p < 0.001

Several biological properties associated with EGF are also related to ERK5 signalling pathway. For example, it is well known that EGF can promote S phase entry in cell cycle in which ERK5 is strictly required in HeLa cells.^17^ Therefore, we studied if sorafenib affects cell cycle progression mediated by EGF. To this end, HeLa cells, arrested in G0/G1 by double‐thymidine block, were exposed to EGF in the presence/absence of sorafenib or XMD8‐92 as indicated in Figure 4A. As it is shown, sorafenib dramatically reduces the entry in S phase triggered by EGF (Figure 4B and 4C).

**FIGURE 5:**
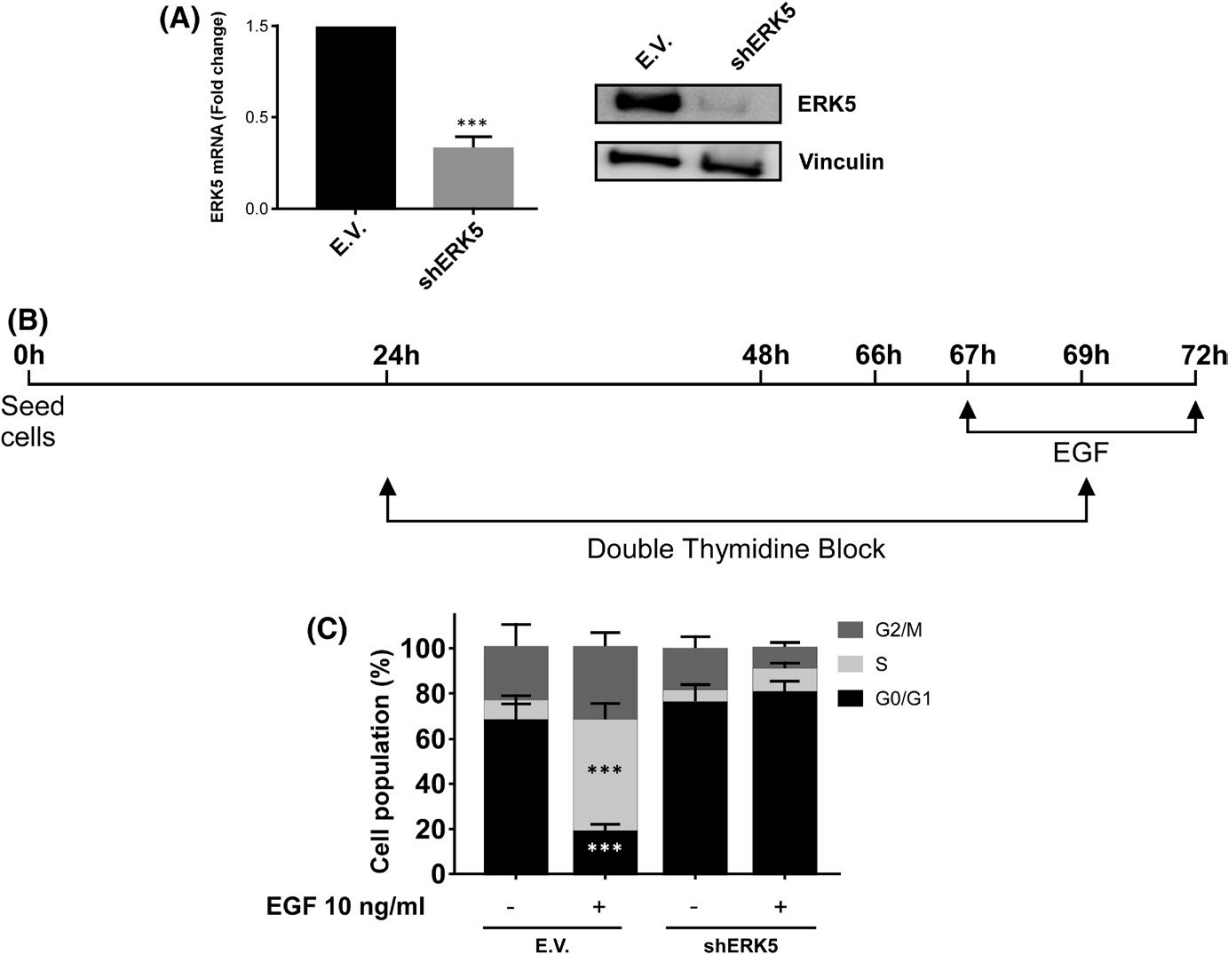
ERK5 is required for EGF‐mediated S phase induction. (A) Right panel: Total RNA from HeLa cells infected with lentiviruses carrying the empty vector pLKO‐puro (E.V.) or pLKO‐shERK5 (shERK5), expressing a specific shRNA for ERK5, were collected and ERK5 mRNA was evaluated by RT‐qPCR. GAPDH was used as an endogenous control. Left panel: Levels of ERK5 were evaluated by Western blot in HeLa E.V. and HeLa shERK5 cell lines. Vinculin was used as a loading control. (B) Timeline of the experiment. HeLa cells infected with (E.V.) or with (shERK5) were treated as in Figure 4A but without exposure to sorafenib or XMD8‐92. After treatment, cells were collected and evaluated by flow cytometry for cell cycle progression. (C) Histogram showing the average of 3 independent experiments representing the percentage of population in the different phases of the cell cycle. Statistics were referred to untreated cells. Bars mean standard deviation. *p < 0.05, **p < 0.01 and ***p < 0.001

Next, HeLa cells with abrogated ERK5 expression by using a specific shRNA (Figure 5A), were exposed to EGF (Figure 5B), showing a lack of response to EGF when compared to control cells in terms of cell cycle progression (Figure 5C), indicating the key role of ERK5 in this biological effect associated with EGF.

**FIGURE 6:**
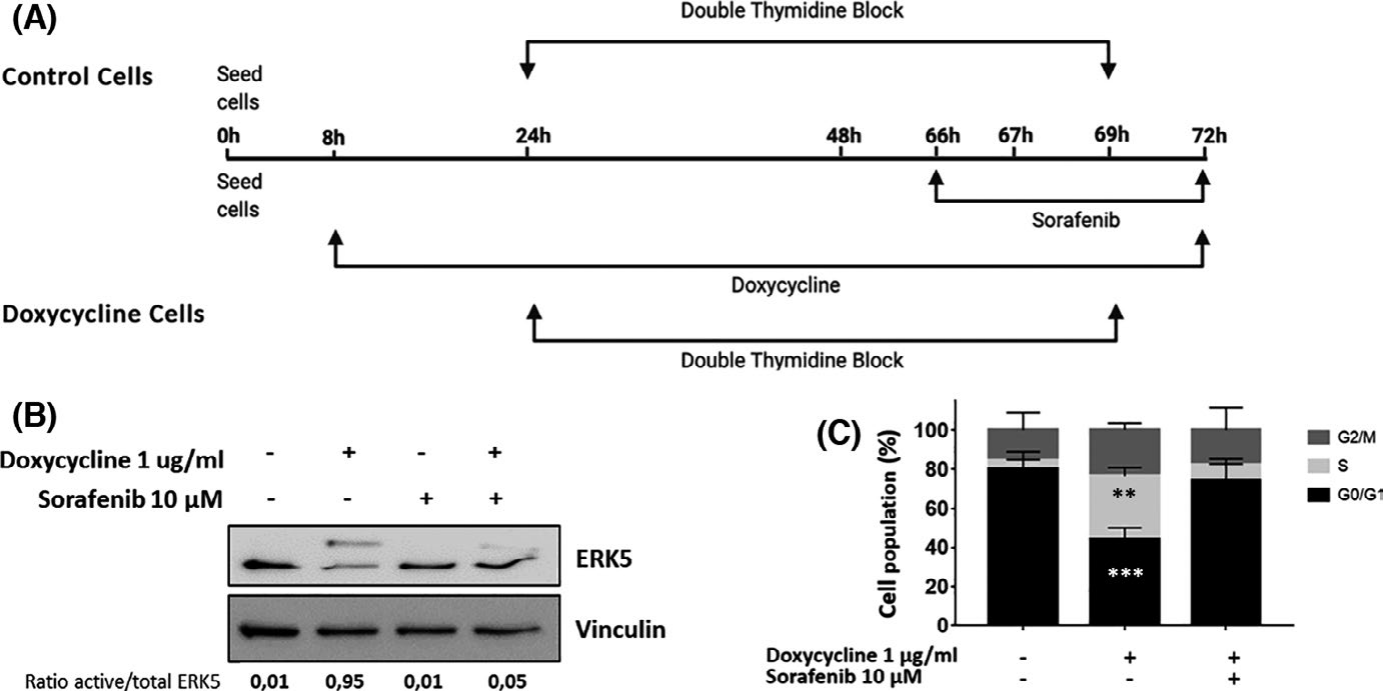
Activation of ERK5 signalling promotes S phase induction that is blocked by sorafenib. (A) Timeline of the experiment. HeLa cells infected with pSLIK MEK5DD‐mRFP1 lentiviruses expressing an inducible hyperactive MEK5 form (MEK5DD) were seeded and 8 hours later, they were incubated in the presence/absence of Doxycycline at the indicated concentration until the end of the experiment. Doxycycline‐treated and untreated cells were exposed to double‐thymidine block and Doxycycline‐treated cells were incubated in the presence/absence of sorafenib as in Figure 4A. Then, cell cycle was evaluated by flow cytometry. (B) HeLa cells were infected with pSLIK MEK5DD‐mRFP1 lentiviruses carrying a Doxycycline‐inducible hyperactivated MEK5 (MEK5DD). Cells were treated with Doxycycline for 24 h at the indicated concentration and for the last 6 h, they were incubated in presence or absence of 10 µM sorafenib. Numbers below blots indicate the ratio between active and total protein. Images show a representative blot out of 3 with nearly identical results. Total cell lysates were processed as described in material and methods and blotted against total ERK5. Vinculin was used as a loading control. (C) Histogram showing the average of 3 independent experiments representing the percentage of population in the different phases of the cell cycle. Statistics were referred to untreated cells. Bars mean standard deviation. *p < 0.05, **p < 0.01 and ***p < 0.001

Finally, we generated an experimental model of HeLa cells with an inducible constitutive active MEK5 (MEK5DD) that was challenged in terms of cell cycle progression (Figure 6A). As it is shown, exclusive activation of MEK5 by Doxycycline treatment (Figure 6B), promotes an increase in S phase entrance, lower than the one triggered by EGF, but that is also abolished by treatment with sorafenib (Figure 6C).

In summary, this set of experiments, demonstrate that sorafenib blocks the cell cycle progression in response to EGF, and this effect is at least partially due to its inhibitory effect exerted onto ERK5 signalling pathway.

### 3.3 Sorafenib blocks migration after EGF stimulation or ERK5 activation

**FIGURE 7:**
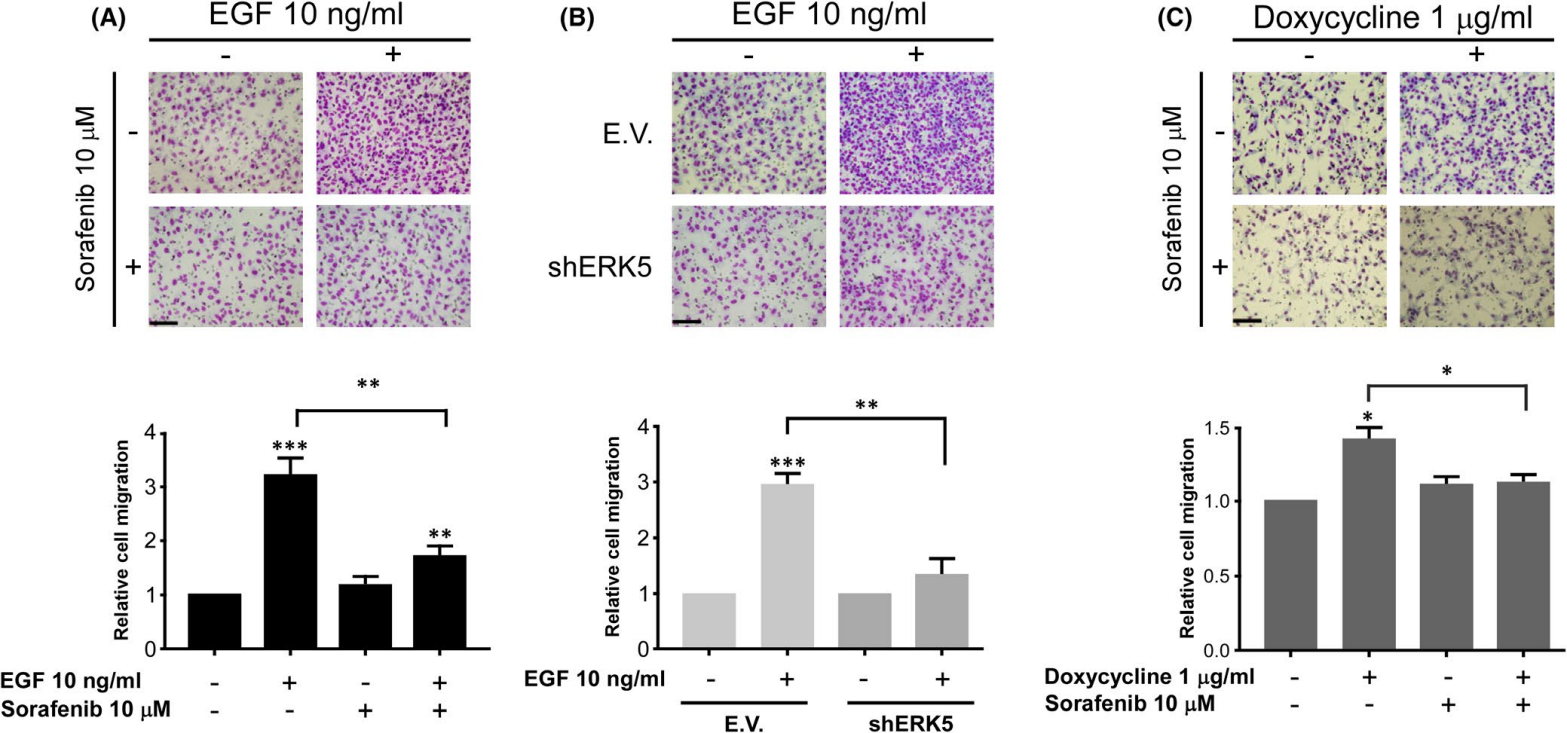
Sorafenib blocks EGF migration in an ERK5‐dependent fashion. (A) Upper panel: Representative image of a transwell migration assay in parental HeLa cells incubated in presence or absence of sorafenib, EGF, or both at the indicated concentrations for 24 h. Magnification ×100 (n = 3). Scale bars represent 100 µm. Lower panel: Histogram showing the average of 3 independent experiments representing the relative migration referred to untreated cells set as 1. Bars mean standard deviation. *p < 0.05, **p < 0.01 and ***p < 0.001. (B) Upper panel: migration assay in HeLa cells infected with lentiviruses carrying either empty vector (E.V) or shRNA specific to ERK5 (shERK5). Cells were incubated in presence or absence of EGF at the indicated concentration for 24 h. Magnification ×100 (n = 3). Scale bars represent 100 µm. Lower panel: Histogram showing the average of 3 independent experiments representing relative migration referred to untreated cells for each condition considered as 1. Bars mean standard deviation. *p < 0.05, **p < 0.01 and ***p < 0.001. (C) Upper panel: Representative images of a transwell migration assay in HeLa cells infected with lentiviruses carrying an inducible MEK5 hyperactive form (MEK5DD). Cells were incubated in presence of Doxycycline at the indicated concentration for 16 h prior to migration assay. After that, cells were seeded onto transwells in the presence or absence of Doxycycline and treated or not with sorafenib at the indicated concentrations for 24 h. Magnification ×100 (n = 3). Scale bars represent 100 µm. Lower panel: Histogram showing the average of 3 independent experiments representing relative migration referred to untreated cells set as 1. Bars mean standard deviation. *p < 0.05, **p < 0.01 and ***p < 0.001

In addition, we analysed the ability of sorafenib to modulate another well‐established property associated with EGF, such as cell migration.^41^ As indicated in Figure 7A, transwell assays showed that EGF promotes a marked increase in HeLa cells migration, which is clearly blocked by the presence of sorafenib. Next, ERK5 knock‐down in Hela cells abrogates the increase in cell migration stimulated by EGF (Figure 7B). Finally, in HeLa cells expressing an inducible active form of MEK5 (MEK5DD), the addition of Doxycycline promotes an increase in migration, lower than the one triggered by EGF, which was also abolished by the presence of sorafenib (Figure 7C). Therefore, all the previous data support that the effect of sorafenib in terms of cell migration could be partially explained by the effect exerted onto ERK5 signalling pathway.

## 4 DISCUSSION

Protein kinases are key players in cell homeostasis through the modification exerted onto the activity of other proteins, thus modulating a great number of cellular processes. Therefore, the deregulation of their activity leads to important pathologies being cancer a paradigmatic example. In this regard, protein kinases have become new targets for cancer therapy and the development of specific inhibitors is a very active field of research. However, the appearance of resistance to chemical inhibitors against protein kinases represents a first‐order scientific challenge because of the health implications that this entails.^42^

From the present work, several conclusions can be obtained. First, and the most obvious, it is the fact that the ERK5 signalling pathway, which has a clear oncogenic potential,^43^ is a novel target of sorafenib. In this regard, although sorafenib was initially described as a specific inhibitor of Raf, later on, it was shown to be a potent inhibitor of other kinases as PDGFR and VEGFR.^44^ Therefore, our data add a novel target to this list, reinforcing the multikinase inhibitor character of sorafenib. The structure of the catalytic domain of the protein kinases obtained from the X‐ray diffraction shows the existence of a N‐ter lobe and a C‐ter lobe that form a cleft which serves as a binding site for ATP and Mg^2^.^45^ Indeed, the vast majority of protein kinase inhibitors have this cavity as a target, and this supposes a problem of specificity and probably contributes to the development of off‐target effects.^42^ Thus, it is easily understandable that various members of the ERK5 signalling pathway can also be inhibited by sorafenib, which would show different affinity for the ATP‐binding site of each member of the pathway. This could be the case of the active MEK5, supporting some type of specificity as this study suggests.

Second, it is important to mention that the blockage of the cellular response associated with EGF by sorafenib seems to be Raf‐independent, at least in our model of HeLa cells. Recently, it has been reported that oncogenic B‐Raf is an activator of ERK5 signalling pathway.^21^ However, several evidences exclude B‐Raf in our experimental model. For example, the lack of effect of sorafenib onto ERK1/2 activation in response to EGF observed in HeLa and U2OS cells. In the case of A549 cells, we detected a marked decrease in ERK1/2 activation by EGF in the presence of sorafenib; however, this experimental model harbours a mutant *K*‐*Ras* ^46^ that could account for this differential behaviour. In addition, the effect observed using a MEK5 constitutively active form (MEK5DD) discards a direct implication of ERK1/2 signalling pathway in our experimental model. In this regard, previous observations described that ERK1/2 activation in response to EGF signalling could be insensitive to sorafenib in HeLa cells,^47^ which could be extrapolated to other experimental models, as we show in U2OS. Indeed, our in silico studies suggest that sorafenib exhibit a preference for active MEK5 that is also demonstrated by using hyperactive MEK5 or in the context of EGFR activation and the subsequent MEK5 activation. This observation, therefore, could give a potential selective character to sorafenib in those tumours with a hyperactive MEK5 due either to genetic alteration in MEK5 or to alterations in the elements of the pathway that render an active MEK5.

Third, our data reveal that ERK5 signalling pathway could be a novel link between EGF signalling and sorafenib. For example, the effects of EGF onto cell cycle and the inhibitory effects exerted by sorafenib could be mediated by ERK5. In this regard, it is known that ERK5 is a key regulator of cell cycle. Indeed, ERK5 has been related to G1/S transition^47^ and mitotic progression.^48^ Interestingly, sorafenib is known to promote G0/G1 arrest^49^ in an opposite way to EGF.^17^ However, this observation needs further studies, especially considering that ERK5 activity could be modulated during mitosis through a CDK‐dependent phosphorylation which renders and inactive ERK5 and also regulates ERK5 subcellular localization.^51^ In addition, it is important to mention that alterations in EGF signalling pathway have been observed in several pathologies being lung cancer a paradoxical example of EGFR targeted therapy.^52^ Indeed, our finding supports previous observations suggesting the use of sorafenib in lung cancer at preclinical^53^ and clinical level.^54^, ^55^ Furthermore, our observations could fit specially with those cases in which alteration in the ERK5 signalling pathway has been shown,^56^ suggesting that ERK5 signalling pathway could be a novel target in lung cancer. However, our observations could also have implications in other types of tumours. For example, it has been reported a critical role for ERK5 signalling pathway in hepatocellular carcinoma (HCC)^57^ and, interestingly, sorafenib is one of the few standard treatments in advanced HCC.^58^ Therefore, our data suggest that maybe some of the therapeutic properties of sorafenib in HCC could be due to its inhibitory effect exerted onto ERK5 signalling pathway.

Finally, our observations open new therapeutic possibilities for sorafenib. In this regard, sorafenib has been reported to exert its therapeutic effect through the inhibition of tumour progression, affecting processes like angiogenesis, epithelial‐to‐mesenchymal transition (EMT) or migration.^59^, ^60^ Interestingly, in these processes, ERK5 has a determinant role.^61^, ^62^, ^63^ Furthermore, downstream targets of ERK5 signalling pathway, as MEF2 transcription factors, which have a critical role related to the oncogenic capacities of ERK5, could be implicated in our observations (for a review see^64^). In fact, MEF2 transcription factors have been related to cell migration and invasion,^65^ angiogenesis^66^ or EMT transition.^67^ Therefore, our data suggest that ERK5, probably through its downstream targets as MEF2, could be one of the mediators for the therapeutic effects of sorafenib onto tumour progression, supporting a wider use of this multikinase inhibitor in different pathologies.

In conclusion, our present report indicates that ERK5 signalling pathway is a novel target of sorafenib opening new opportunities for the therapeutic use of this drug.

## CONFLICT OF INTEREST

The authors declare no conflict of interest.

## AUTHOR CONTRIBUTIONS

**Marta Ortega‐Muelas:** Investigation (lead); Writing‐review & editing (lead). **Olga Roche:** Investigation (lead); Writing‐original draft (lead); Writing‐review & editing (supporting). **Diego M. Fernandez‐Aroca:** Investigation (lead); Writing‐review & editing (lead). **Jose A Encinar:** Formal analysis (lead); Methodology (lead); Software (lead); Writing‐review & editing (equal). **David Albandea Rodriguez:** Investigation (supporting); Writing‐review & editing (supporting). **Elena Arconada‐Luque:** Investigation (supporting); Writing‐review & editing (supporting). **Raquel Pascual‐Serra:** Investigation (supporting); Writing‐review & editing (supporting). **Ismael Muñoz:** Investigation (supporting); Writing‐review & editing (supporting). **Isabel Sanchez‐Perez:** Investigation (supporting); Writing‐review & editing (supporting). **Borja Belandia:** Investigation (supporting); Writing‐review & editing (supporting). **Maria j Ruiz‐Hidalgo:** Investigation (supporting); Writing‐review & editing (supporting). **Ricardo Sanchez‐Prieto:** Conceptualization (lead); Funding acquisition (lead); Supervision (lead); Writing‐original draft (lead).

## Supporting information

Fig S1

Fig S2

Supplementary Material

## Data Availability

The data that support the findings of this study are available from the corresponding author upon reasonable request.
