# Supplementary material for: ERK5 signalling pathway is a novel target of sorafenib: Implication in EGF biology
Source: J Cell Mol Med. 2021 Oct 16;25(22):10591–603. doi: 10.1111/jcmm.16990 (PMC8581332; doi:10.1111/jcmm.16990)
Supplement: Supplementary file 1 — Fig S1 [file JCMM-25-10591-s003.pdf]

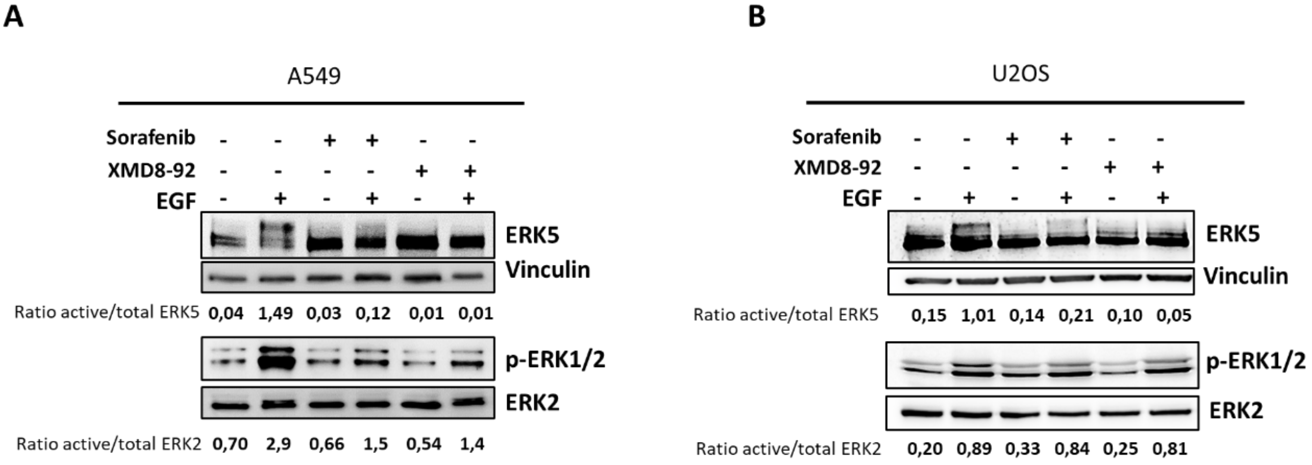

Supplementary Figure 1. Sorafenib blocks ERK5 activation mediated by EGF in A549 and U20S. A) Sub-confluent cultures of A549 cells were exposed to Sorafenib (10µM), XMD8-92 (10µM) for 30 minutes and then exposed for 15 minutes EGF 2 ng/ml. Then total cell lysates were collected and protein extracts (60 µg for ERK1/2 or 120 µg for ERK5) were blotted against the indicated antibody. Vinculin was used as a loading control. B) Sub-confluent cultures of U2OS were treated and processed as in A). Number below blots indicates the ratio between active and total protein, Images show a representative blot out of 3 with nearly identical results.
