# Supplementary material for: ERK5 signalling pathway is a novel target of sorafenib: Implication in EGF biology
Source: J Cell Mol Med. 2021 Oct 16;25(22):10591–603. doi: 10.1111/jcmm.16990 (PMC8581332; doi:10.1111/jcmm.16990)
Supplement: Supplementary file 2 — Fig S2 [file JCMM-25-10591-s002.pdf]

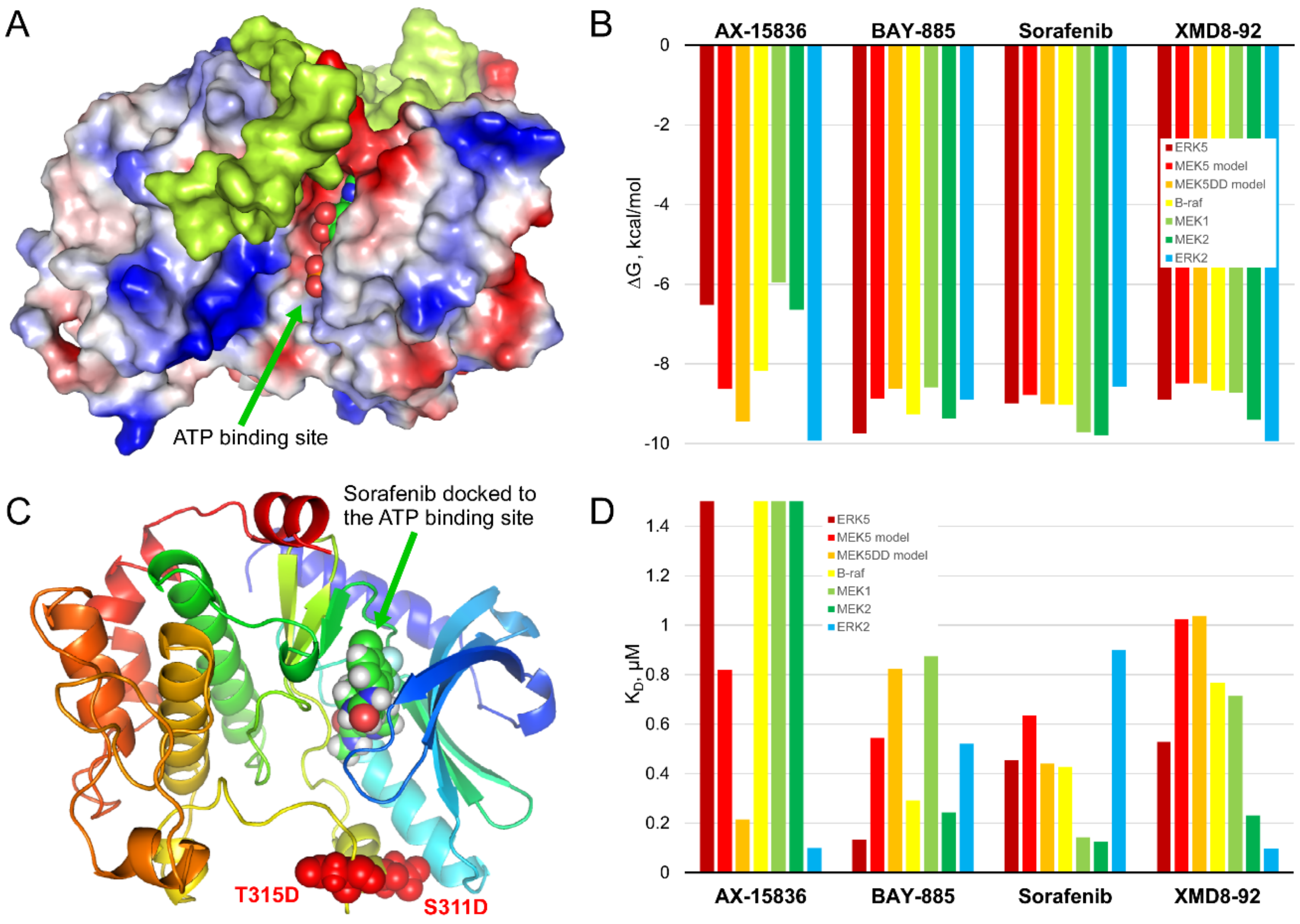

Supplementary Figure 2. Molecular docking simulations of different compounds with inhibitory activity on various protein kinases. A) 3D structure of the catalytic domain of ERK5 kinase (isopotential surface in red for acidic amino acids, blue for basic amino acids and gray/white for the others), interacting with the C-terminal domain of the MKK5 protein-kinase (lemon color). At the ATP binding site there is an ADP molecule represented as spheres (4IC7). B) Gibbs free energy variation values (ΔG, kcal/mol) calculated from the molecular docking simulations of different compounds to the ATP binding site of the catalytic domain of each protein kinases. C) Secondary structure of the catalytic domain of MKK5-S311D, T315D modeled using the 3ZLS structure of MEK1 as a template. The location of the Asp311 and Asp315 residues is indicated by a representation of red spheres. The structure of Sorafenib (spheres with green carbons) is located at the ATP binding site and has been calculated from molecular docking simulations. Panel D shows the calculated values (KD = expΔG/RT) of the dissociation constants, KD [μM], of different compounds using the ΔG data shown in panel B. The panels A and C have been prepared with PyMol 2.0 software.
