# Supplementary material for: ERK5 signalling pathway is a novel target of sorafenib: Implication in EGF biology
Source: J Cell Mol Med. 2021 Oct 16;25(22):10591–603. doi: 10.1111/jcmm.16990 (PMC8581332; doi:10.1111/jcmm.16990)
Supplement: Supplementary file 3 — Supplementary Material [file JCMM-25-10591-s001.docx]

**Supplementary Materials:**

**Supplementary Figure 1. Sorafenib blocks ERK5 activation mediated by EGF in A549 and U2OS cells.**

**Supplementary Figure 2. Molecular docking simulations of different compounds with inhibitory activity on various protein kinases.**
